# Investigation of the hydrodynamic characteristics of an axial flow pump system under special utilization conditions

**DOI:** 10.1038/s41598-022-09157-1

**Published:** 2022-03-25

**Authors:** Xiaowen Zhang, Fangping Tang

**Affiliations:** College of Hydraulic Science and Engineering, Yangzhou University, Yangzhou, 225009 China

**Keywords:** Energy infrastructure, Mechanical engineering, Renewable energy

## Abstract

In actual operation, axial flow pump stations are often used for various special purposes to meet changing needs. However, because the hydrodynamic characteristics of axial flow pump systems are still unclear when used for special purposes, there are many risks when pump systems are used for special purposes. To explore the hydrodynamic characteristics of an axial flow pump system under special utilization conditions, a high-precision full-feature test bench for an axial flow pump system is established in this paper. For the first time, an energy characteristics experiment and a pressure fluctuation measurement for a pump are carried out for a large axial flow pump system model under zero head, reverse pump and reverse power generation conditions. Then, ANSYS CFX software is used to solve the continuous equation and Reynolds average Navier–Stokes equation, combined with the SST *k–ω* turbulence model, and the characteristic curve and internal flow field of the pump system under special conditions are obtained. Finally, the numerical simulation results are compared with the experimental results. The results show that the velocity gradient distribution in the pump is uniform under the near zero head condition (NZHC), and there is no obvious flow collision and reflux phenomenon in the pump. Compared with the designed condition (DC), the peak-to-peak value (PPV) of pressure pulsation at the inlet of the impeller decreased by 67.16%, and the PPV at the outlet of the impeller decreased by 8.14% at *H* = 0 m. The maximum value of the main frequency amplitude (MFA) in the impeller area appears at the impeller inlet. Under reverse pump conditions (RPC), the phenomenon of unstable flow in the pump system is obvious, and a large range of recirculation zones appears in the nonworking face of the blade. Compared with the DC, the PPV of the impeller inlet at the optimal point of RPC increased by 122.61%, and the impeller outlet PPV increased by 11.37%. The maximum value of MFA in the impeller area appears at the impeller inlet. Under the reverse power generation condition (RPGC), no obvious flow separation was found in the nonworking face of the impeller. Compared to the DC, the PPV of the impeller inlet at the optimal point of the RPGC increased by 65.34%, and the PPV of the impeller outlet increased by 206.40%.

## Introduction

In recent years, a large number of large axial flow pump stations have been built around the world, especially in China. Axial flow pump stations are characterized by low heads and large flows and are often located along rivers and coastal areas. The fluctuation of the water level in the upper and lower reaches is very large, which means that axial flow pump systems must cope with the changeable demands in actual operation. For example, when the upstream and downstream water level difference is very small, an axial flow pump system must be used for nearly zero head drainage^1,2^. When the upstream water level is higher than the downstream water level, the axial flow pump system may need to carry out reverse water lifting^3^, and the axial flow pump system can also be used to generate electricity from the upstream residual water^4,5^.

Although the application conditions of axial flow pump stations are continuously expanding, the main research results thus far have still concentrated on the hydrodynamic characteristics of conventional pump conditions^6–8^. There are few studies on the hydrodynamic characteristics of axial flow pump systems under special utilization conditions. The hydrodynamic characteristics of axial flow pump systems under special utilization conditions are still unclear, and there may be many risks, such as unit vibration and blade fracture, in the special utilization of the pump system.

Zero-lift drainage, reverse pumping and reverse power generation are the most common special uses of large axial flow pump systems. In recent years, scholars have conducted preliminary studies on the hydrodynamic characteristics of axial flow pump systems under these special utilization conditions. Wang et al.^1^ took an inclined axial flow pump system as the research object and carried out numerical calculations and field tests for the first time for an axial flow pump system under near zero head conditions. It was found that the flow pattern in the pump is not particularly chaotic when the inclined axial flow pump system is operated near a zero head, and the hydraulic loss of the guide vane represents the main hydraulic loss of the system. Li et al.^2^ carried out experimental and numerical studies on the vibration of a horizontal axial flow pump system under near zero head conditions and compared and analysed the influencing factors of the vibration of the pump system under designed head and zero head conditions. Ma et al.^3^ studied the hydrodynamic characteristics of an axial flow pump station under reverse pump conditions. It was found that the flow corresponding to the highest efficiency point under reversed pumping conditions shifted, and the hydraulic efficiency decreased significantly. Bozorg et al.^9^ used computational fluid dynamics methods and experimental tests to obtain the energy characteristic curve of a small axial flow pump in reverse power generation operation and noted that the axial flow pump can be used as a turbine to reverse power generation in a low-head Pico hydropower plant. Qian et al.^10^ studied two pump and turbine modes for a small axial flow pump and found that the flow pattern in the pump is better when the small axial flow pump performs reverse power generation, and the reverse power generation condition has a wider high efficiency zone compared to the forward pump condition.

In recent years, an increasing number of researchers have noted that the pressure pulsation inside hydraulic machinery is one of the most important factors affecting the safe and stable operation of hydraulic machinery systems^11–13^, and it should also be the main component of any hydrodynamic characteristics analysis of hydraulic machinery^14^. However, the current research on the hydrodynamic characteristics of axial flow pump systems under special utilization conditions is still scattered, and the research mainly focuses on the energy characteristics of pump systems under special utilization conditions^15–17^. There is a lack of in-depth comparison and discussion on the hydrodynamic characteristics of pump systems, especially the pressure fluctuation in pumps. This also leads to an ineffective judgement the safety and stability of the pump system under special utilization conditions.

The remainder of this paper is organized as follows: the hydraulic model of the axial flow pump system used in the experiment is introduced in the “Research object” section. In the “Experiment system” section, the specific parameters of the experimental system and the experimental test method are introduced. In the “Experiment results and analysis” section, this paper analyses the propagation law of the pressure pulsation in the pump of the axial flow pump system under special operating conditions from the two aspects of the time domain and the frequency domain, and the pressure pulsation characteristics of the pump system under conventional pump conditions and special operating conditions are compared in detail. In the “Numerical simulation” section, the numerical method and solution scheme for the axial flow pump under special utilization conditions are introduced. In “Numerical simulation results and analysis”, combined with the flow field in the pump obtained by numerical simulation, this paper further analyses and explains the hydrodynamic characteristics of the pump system under special working conditions, especially the pressure pulsation characteristics in the pump. The “Conclusion” section summarizes the whole work and gives the potential research issues for future research focus.

## Research object

**Figure 1:**
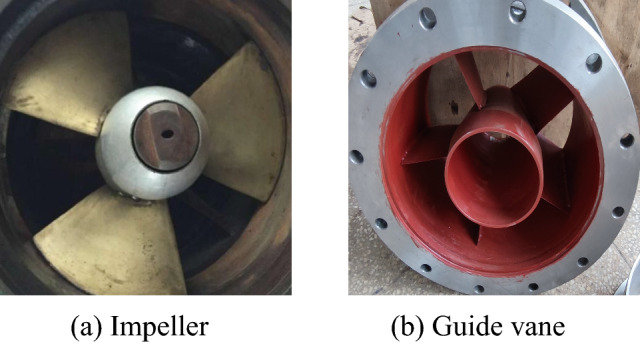
Hydraulic model of the axial flow pump.

**Figure 2:**
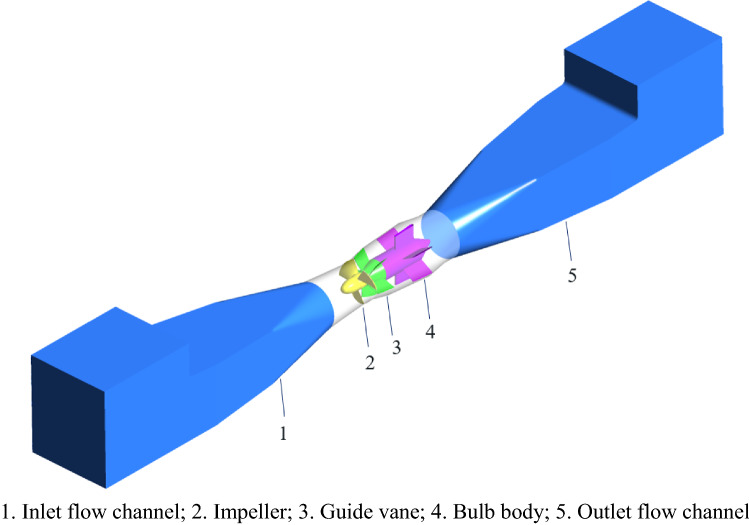
Three-dimensional schematic diagram of the model pump system structure.

**Figure 3:**
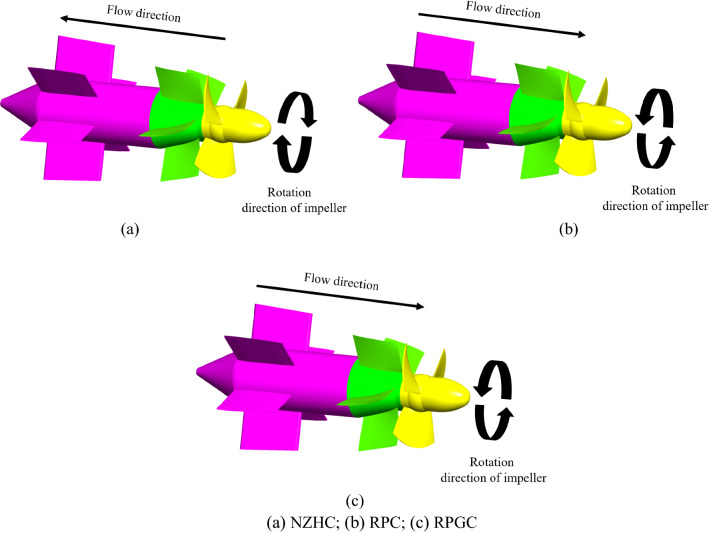
Rotation direction of the impeller under three special utilization conditions.

The hydraulic model of the axial flow pump selected for this experiment is shown in Fig. 1. A schematic diagram of the three-dimensional structure of the model pump system is shown in Fig. 2. To express clearly and concisely, the design condition of the pump system is defined as DC, the near zero head condition is defined as NZHC, the reverse pump condition is defined as RPC, and the reverse power generation condition is defined as RPGC. The rotation direction of the impeller under three special utilization conditions is shown in Fig. 3. In addition, Table 1 shows the main geometric parameters of the model pump system. In addition, Table 1 shows the main parameters of the pump system. The “Specific speed” in Table 1 refers to the speed of the pump impeller when the head is 1 m, the effective power is 1 HP (0.7355 kW) and the flow rate is 0.075 m^3^/s. The calculation formula of the specific speed in Table 1 is as follows:

1 $$n_{s} = \frac{3.65n\sqrt Q }{{H^{\frac{3}{4}} }}$$

where *n* is the rated speed of the pump, r/min. *Q* is the rated flow of the pump, m^3^/s. *H* is the head of the pump, m.

**Table 1:** Main geometric parameters of the pump system model.

Parameter	Symbol	Numerical value
Designed flow rate	*Q*_*d*_	203 l/s
Designed head	*H*_*d*_	2.01 m
Designed efficiency	*η*_*d*_	68.39*%*
Specific speed	n_s_	1179 r/min
Tip clearance	*C*	0.15 mm
Impeller diameter	*D*	300.00 mm
Impeller hub diameter	*d*	94.86 mm
Impeller inlet diameter	*D*_*1*_	293.94 mm
Impeller outlet diameter	*D*_*2*_	293.94 mm
Blade placement angle	*β*_*1*_	0
Impeller blades number	Z_1_	3
Guide vane number	Z_2_	5

## Experimental system

**Figure 4:**
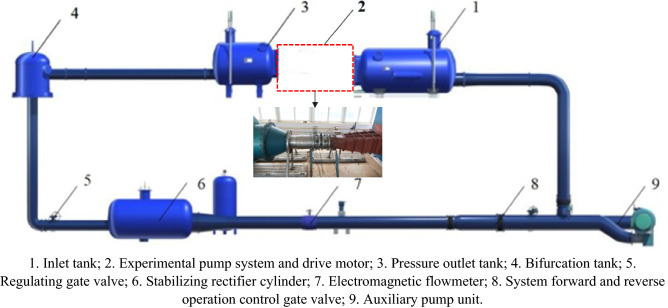
Schematic diagram of the experimental system.

The experiment was conducted on a high-precision hydraulic machinery test bench at the Jiangsu Provincial Key Laboratory of Hydraulic Power Engineering, Yangzhou University. The test bench is a vertical closed circulation system. A schematic diagram of the experimental system is shown in Fig. 4.

**Figure 5:**
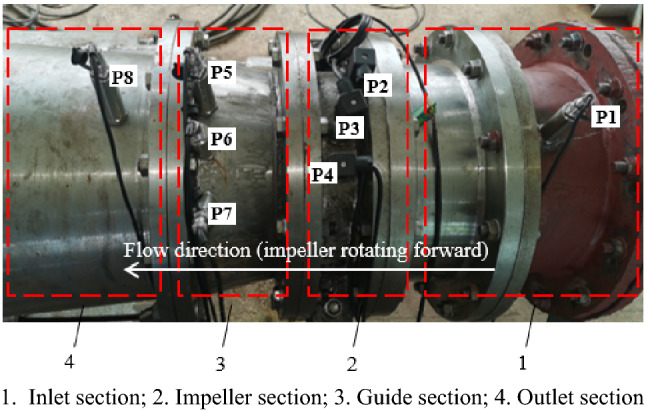
Measurement of pressure fluctuation in the experiment.

**Figure 6:**
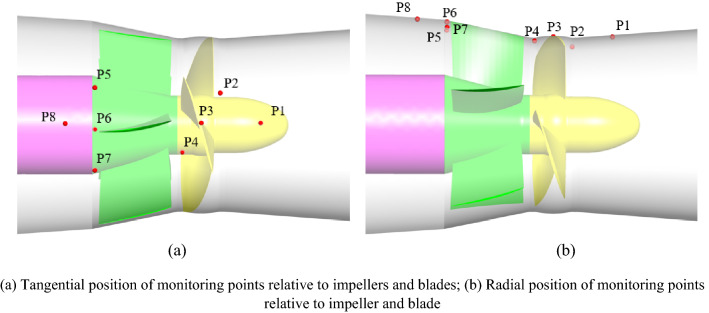
Schematic diagram of the specific locations of the monitoring points arranged in the experiment.

The main instruments of the test measurement system include a differential pressure transmitter, electromagnetic flowmeter, speed torque sensor and absolute pressure transmitter. The basic parameters of the instrument are shown in Table 2. The comprehensive error of the test system is ± 0.39%. The test process is strictly in accordance with the requirements of the acceptance test procedure for the pump model and device model (SL 140-2006). In this experiment, eight pressure pulsation measuring points were arranged in the inlet section (monitoring point P1), impeller inlet (monitoring point P2), impeller middle (monitoring point P3), impeller outlet (monitoring point P4), guide vane outlet (monitoring point P5, P6, P7) and outlet section (monitoring point P8). Figure 5 shows the measurement diagram of pressure fluctuation in the experiment. Figure 6 shows the specific locations of the monitoring points in the experiment. A CY200 high-frequency dynamic microsensor was used in the pressure pulsation test. The sampling frequency of the sensors at monitoring points P2, P3, and P4 in the impeller area was 3 kHz, and the sampling frequency of the sensors at the other monitoring points was 1 kHz. The voltage output was 0 ~ 5 V, and the accuracy level was 0.1%. A 485-20 concentrator matched with the sensor was used for the acquisition instrument. The uncertainty of the test system has an important influence on the test results. The system uncertainty of the pump performance test is the square and root of each single system uncertainty. The calculation formula is as follows^19^:

2 $$\left( {E_{\eta } } \right)_{s} = \pm \sqrt {E_{Q}^{2} + E_{H}^{2} + E_{M}^{2} + E_{n}^{2} } = \pm 0.274\%$$

where *E*_*Q*_ is the system uncertainty of the flow measurement and the calibration result is ± 0.2%. *E*_*H*_ is the uncertainty of the static head measurement system, and the calibration results are in the full range of ± 0.10%. *E*_*M*_ is the system uncertainty of the torque measurement, and the uncertainty of the torque speed sensor is ± 0.15%. *E*_*n*_ is the system uncertainty of the speed measurement. When the sampling period is 2 s and the speed is not less than 1000 r/min, the uncertainty is ± 0.05%.

**Table 2:** Main instruments of the experimental measurement system.

Measuring items	Instrument name	Instrument types	Instrument range	Calibration accuracy (%)
Head	Difference pressure transmitter	EJA 110A	0 ~ 200 kPa	± 0.1
Flow	Electromagnetic flowmeter	E-mag type	DN400 mm	± 0.20
Torque	Speed and torque sensor	ZJ	200 N m	± 0.15

## Experimental results and analysis

### Experimental results of external characteristics

**Figure 7:**
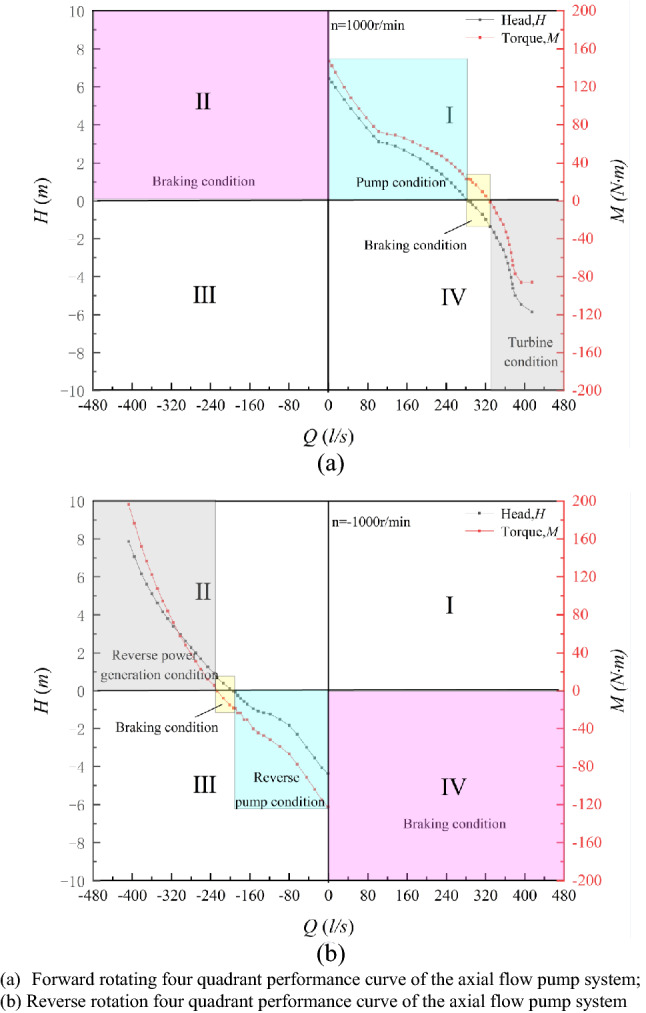
Four quadrant performance curves obtained from the axial flow pump system experiment.

**Figure 8:**
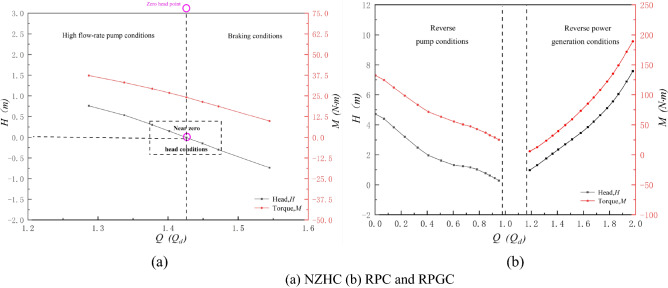
Local amplification diagram of the energy characteristic curve of the axial flow pump system under three special utilization conditions.

The special operation condition test of the axial flow pump system includes positive rotation near the zero head condition, reverse pump condition and reverse power generation condition. The speed of the impeller under three special utilization conditions is 1000 r/min. To show the specific position of the special working condition of the axial flow pump system in the full working condition curve of the axial flow pump system, Fig. 7 shows the four-quadrant performance curve obtained by the axial flow pump system experiment. Figure 8 shows a partially enlarged view of the energy characteristic curves of the axial flow pump system under three special utilization conditions. The flow *Q* in Fig. 8 is dimensionlessly processed as follows^19^:

3 $$Q = \frac{{Q_{i} }}{{Q_{d} }}$$

where *Q*_*i*_ is the flow rate of the pump system under the *i* working condition and *Q*_*d*_ is the flow rate of the pump system under the design condition.

Table 3 shows the external characteristic parameters of the key operating points under special utilization conditions. According to Table 3, compared to the DC, the flow rate at the zero head point increased by 42.00%, and the torque was 22.97 N m, which decreased by 58.21%. The flow rate of the RPC optimum point decreased by 15.00%, the head was 0.76 m, decreased by 61.22%, and the torque was 37.07 N m, a decrease of 32.56%. The flow rate of the RPGC optimum point increased by 62.01%, the head was 3.85 m, increased by 96.00%, and the torque was 85.12 N m, an increase of 55.00%.

**Table 3:** External characteristic parameters of key operating points under special utilization conditions.

Measuring items	Flow rate *Q*/*Q*_*d*_	Head *H*/*m*	Torque *M*/N m	Shaft power *p*/kW
DC	1.00	1.96	54.96	5.76
Zero head point	1.42	0.00	22.97	2.41
RPC optimum point	0.82	0.76	37.07	3.88
RPGC optimum point	1.62	3.85	85.12	8.92

### Time and frequency domain analysis of the pressure pulsation signal

To eliminate interferences such as static pressure, the instantaneous pressure collected in the test (4 impeller rotation cycles) is dimensionless, and the pressure coefficient *C*_*p*_ is used to characterize the pressure fluctuation amplitude. The formula is as follows^20^:

4 $$C_{p} = \frac{{p - \overline{p} }}{{0.5\rho u_{2}^{2} }}$$

where *p* is the transient pressure value, $\overline{p}$ is the average pressure value, and *u*_2_ is the circumferential velocity of the impeller outlet.

At the same time, to capture the detailed characteristics of the pressure pulsation signal, a fast Fourier transform (FFT) is used to transform the pressure pulsation signal. In the pressure pulsation spectrum, the X-axis is the frequency multiple, the Y-axis is the monitoring point, and the Z-axis is the dimensionless pressure pulsation amplitude. The formula for the frequency conversion multiple is as follows^20^:

5 $$N_{F} = \frac{f}{{f_{n} }} = \frac{60F}{n}$$

where *F* is the frequency after Fourier transform and *n* is the impeller speed.

#### Near zero head condition

**Figure 9:**
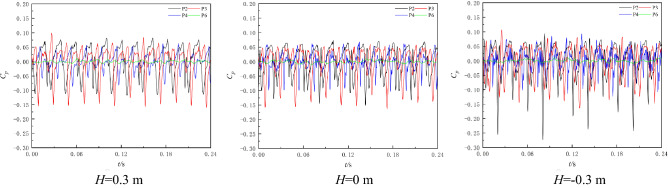
Time-domain diagram of pressure fluctuation under the NZHC.

In this section, the experimental data of *H* = 0.3 m, *H* = 0 m, and *H* = − 0.3 m are selected to analyse the pressure pulsation characteristics of the NZHC. Figure 9 shows the pressure fluctuation time-domain diagram of each monitoring point. Figure 9 shows that when the pump system runs near the zero head, the pressure pulsation regularity of each monitoring point is still good. By observing the pressure pulsation waveforms under different working conditions, it is found that in an impeller rotation cycle, three main peaks and three main troughs can be observed at each monitoring point in the impeller area, indicating that the pressure pulsation in the impeller area is still dominated by the number of blades when the pump system operates near the zero head. This is also consistent with the phenomenon found by Wang et al.^1^ in on-site measurements of pressure fluctuations of an axial flow pump station under an NZHC^1^. When the head of the pump system gradually tends to be negative from positive, the shape of the peak and trough becomes gradually sharp. In a rotating period of the impeller, the number of secondary peaks carried by the single main wave of monitoring point P4 at the outlet of the impeller increases, the difference between the main peak and the secondary peak gradually decreases, and the occurrence of the secondary peak has no obvious regularity. Compared with the impeller outlet measuring point, the number of secondary peaks carried by each main peak of impeller inlet measuring point P2 and impeller middle measuring point P3 has no obvious increase.

**Figure 10:**
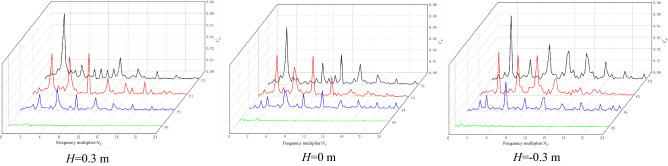
Frequency domain diagram of pressure fluctuation under the NZHC.

Figure 10 shows the frequency domain diagram of pressure fluctuation at each monitoring point under the NZHC. The following conclusions can be obtained from Fig. 9. First, the main frequency of pressure fluctuation at impeller inlet monitoring point P2 and impeller middle monitoring point P3 is the blade rotation frequency (BPF), and the main frequency of pressure fluctuation at impeller outlet monitoring point P4 is twice the blade frequency (2BPF). Second, by observing the pressure fluctuation waveform of P6 at the outlet of the guide vane, it is found that the main frequency of pressure fluctuation at *H* = 0.3 m and *H* = 0 m is the impeller rotation frequency, which indicates that the outlet of the guide vane is greatly affected by impeller rotation under the NZHC. Third, the pressure fluctuation at *H* = − 0.3 m is dominated by a low-frequency signal, and the main frequency of pressure fluctuation is the axial rotation frequency (SF). This shows that under the condition of a negative head, the influence of impeller rotation on the outlet of the guide vane is weak, and the axial rotation frequency has occupied the leading role.

#### Reverse pump condition

**Figure 11:**
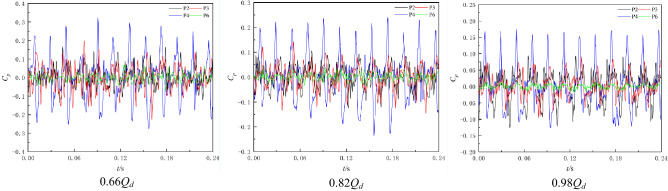
Time-domain diagram of pressure fluctuation under RPC.

When the pump system is under the RPC, the pump system enters the reverse operation state, and the water flow direction in the pump system is opposite to the forward pump condition. At this time, the impeller inlet is transformed into the impeller outlet under reverse operation, and the impeller outlet is transformed into the impeller inlet under reverse operation. The guide vane is transformed from the outlet water component after the impeller outlet to the inlet water component before the impeller inlet. Through the RPC experiment, it is found that the optimum RPC flow condition is 0.82*Q*_*d*_. Therefore, this section selects three typical flow conditions of 0.8 times (0.66*Q*_*d*_), 1.0 times (0.82*Q*_*d*_) and 1.2 times (0.98*Q*_*d*_) the optimum point flow for analysis. Figure 11 shows the time-domain diagram of the pressure fluctuation of each monitoring point. Figure 11 shows that since the impeller is designed for positive operation, when the pump system is under the RPC, there are serious backflow, vortex and other unstable flow phenomena in the pump, and the pressure pulsation signal of each monitoring point is relatively complex. In an impeller rotation cycle, three obvious peaks and three troughs can be observed at impeller inlet monitoring point P4 under different flow conditions. The difference between the main wave peak and the secondary wave peak of monitoring point P2 at the impeller outlet and monitoring point P3 at the middle of the impeller under reverse operation is small. Monitoring point P6 has multiple main peaks and valleys in one impeller rotation cycle. Comparing the pressure pulsation of each monitoring point under different flow conditions, it can be found that under different flow conditions, each main wave peak of monitoring points P2 and P3 has multiple secondary peaks, and the occurrence of secondary peaks has no obvious regularity. Compared with monitoring points P2 and P3, the periodic law of pressure fluctuation at P4 is better, and the pressure fluctuation signal component is relatively simple.

**Figure 12:**
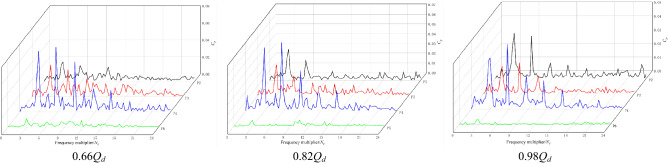
Frequency domain diagram of pressure fluctuation under the RPC.

Figure 12 shows the frequency domain diagram of the pressure fluctuation of each monitoring point under the RPC. Figure 12 shows that the main frequency of pressure pulsation of each monitoring point in the impeller area under different flow conditions is still dominated by the high-order harmonics of blade frequency and blade frequency. The main frequency of the pressure fluctuation of monitoring point P2 at the impeller outlet is the blade frequency. The main frequency of pressure fluctuation at impeller inlet monitoring point P4 is twice the blade frequency. In the pressure pulsation spectrum of monitoring point P6, the signal component is relatively simple, but the obvious blade frequency component can still be seen under different flow conditions, indicating that the impeller as a pulsation excitation source has a great influence on the upstream flow field under the RPC.

When the pump system is under the RPC, the frequency band of each monitoring point is wide, and there is a large pulsation in the high frequency region. A certain amount of pulsation can still be observed at the monitoring points in the impeller area from 3 to 7BPF. The reason may be that the pump system is in the reverse pump condition, and the impeller inlet reflux and other adverse flow intensify, resulting in water flow on the blade and channel wall caused by different degrees of impact and the formation of different frequencies of pressure waves. Under different flow conditions, the pressure pulsation amplitude of monitoring point P6 is small. With increasing flow rate, the pressure pulsation signal component of monitoring point P6 becomes simpler, and the pulsations in the low-frequency region and high-frequency region disappear, indicating that the inlet flow pattern of the impeller under RPC gradually improves with increasing flow rate.

#### Reverse power generation condition

**Figure 13:**
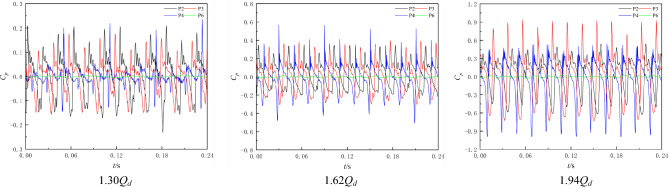
Time domain diagram of pressure fluctuation under the RPGC.

When the pump system is under the RPGC, the pump system enters the reverse operation state, and the flow direction in the pump system is the same as that in the reverse pump condition. Through the RPGC experiment, it is found that the optimum point of the reverse power generation condition is the 1.62*Q*_*d*_ flow condition. Therefore, this section selects three typical flow conditions of 0.8 times (1.30*Q*_*d*_), 1.0 times (1.62*Q*_*d*_) and 1.2 times (1.94*Q*_*d*_) the optimum point flow for analysis. Figure 13 shows the time domain diagram of the pressure fluctuation at each monitoring point under the RPGC. Compared with the RPC, the pressure fluctuation coefficient of each monitoring point increases, but the composition of the pressure fluctuation signal is obviously simple. The phenomenon that each main wave peak of the monitoring point in the impeller area has multiple secondary wave peaks disappears. Under the reverse power generation condition, the occurrence of the secondary wave peak of the monitoring point begins to show obvious regularity and periodicity, and each main wave peak has a fixed secondary peak.

**Figure 14:**
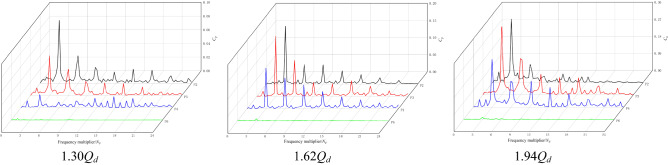
Frequency domain diagram of pressure fluctuation under RPGC.

Figure 14 shows the frequency domain diagram of the pressure fluctuation of each monitoring point under the RPGC. Figure 14 shows that the main frequency of pressure pulsation at the monitoring points in the impeller area under different flow rates is the rotational frequency of the impeller, and the secondary main frequency is basically twice the rotational frequency of the impeller. This shows that the pressure pulsation in the impeller area is still dominated by the number of blades under the RPGC. Monitoring point P6 is far from the impeller, and the pressure pulsation amplitude is significantly smaller due to the relatively small influence of pump rotation. However, the blade frequency component can still be observed under the two flow conditions of 1.62*Q*_*d*_ and 1.94*Q*_*d*_.

By comparing the pressure pulsation frequency domain diagram of each monitoring point under three flow conditions, it is found that the pressure pulsation of the monitoring point in the impeller area is relatively large in the high-frequency region under the flow conditions of 1.30*Q*_*d*_ and 1.94*Q*_*d*_, and the pulsation in the high-frequency region is relatively small under the flow condition of 1.62*Q*_*d*_. The composition of the pressure pulsation signal is also simple, mainly concentrated in the high-order harmonic frequency of the blade frequency. This may be because the 1.62*Q*_*d*_ flow condition is the optimum point of the pump system under the RPGC, the adverse flow in the pump is reduced, and the energy conversion of the pump under the turbine condition is relatively stable. By comparing the pressure fluctuation of different monitoring points, it is found that the occurrence of high-order harmonics of blade frequency has a certain regularity. For example, under the same flow condition, especially in the 1.62*Q*_*d*_ flow condition, the harmonic component appears near the impeller inlet, and the harmonic component also appears in the middle and outlet of the impeller.

### Main frequency amplitude of pressure pulsation

**Figure 15:**
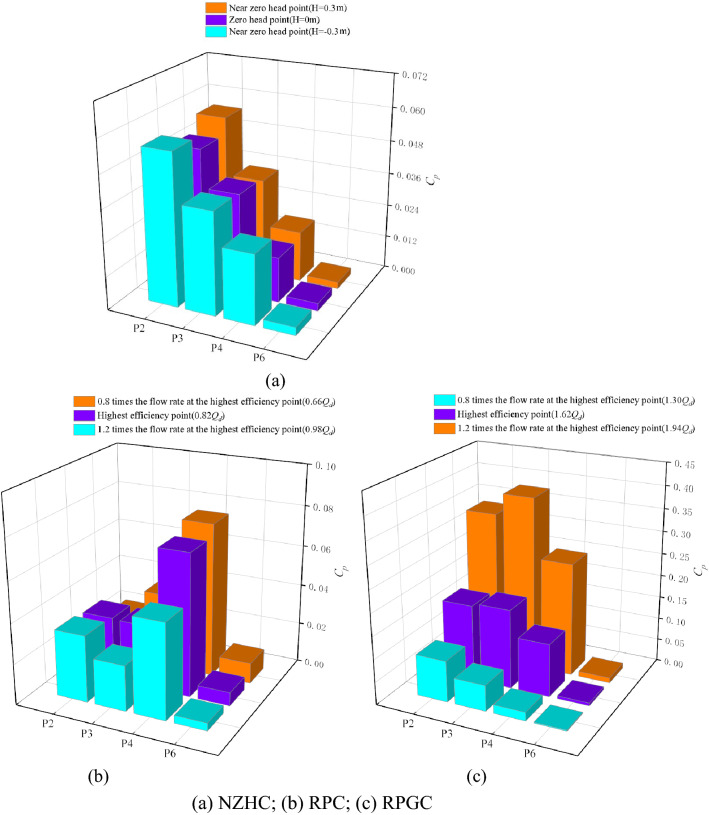
Main frequency amplitudes of monitoring points under special utilization conditions.

In this section, the main frequency amplitude of pressure fluctuation signals at each monitoring point under special working conditions is measured, as shown in Fig. 15. To simplify the expression, the amplitude of the main frequency is referred to as the MFA. It should be noted that the MFAs of monitoring points P2, P3 and P4 measured by experiments under the DC are 0.136, 0.099 and 0.049, respectively.

Figure 15a shows that when the pump system is running near a zero head, the fluid flows from the impeller inlet, flows through the impeller outlet, and finally flows out from the guide vane outlet. The MFA of each monitoring point decreases gradually along the path, and the maximum value of the MFA in the impeller area always appears at the impeller inlet monitoring point P2. When *H* = 0.30 m, *H* = 0 m and *H* =  − 0.30 m, the maximum MFAs are 0.056, 0.050 and 0.055, respectively. The minimum value of the MFA in the impeller area always appears at P4 of the impeller outlet monitoring point. The minimum MFAs of *H* = 0.30 m, *H* = 0 m and *H* =  − 0.30 m are 0.018, 0.016 and 0.025, respectively. Compared with the DC, the MFA of monitoring points P2, P3 and P4 decreased by 62.99%, 63.00% and 66.86%, respectively, when *H* = 0 m.

Figure 15b shows that when the pump system is under the RPC, except for monitoring point P2, the MFA of the monitoring point decreases with increasing flow rate. The maximum value of the MFA in the impeller region always appears at impeller inlet monitoring point P4, which is consistent with the conclusion drawn by Ma et al.^3^ after studying the pressure fluctuation of a bidirectional pump during reverse operation. At a 0.66*Q*_*d*_ flow rate, the minimum value of the MFA in the impeller region appears at P2 of the impeller outlet monitoring point, and the MFA is 0.023. At 0.82*Q*_*d*_ and 0.98*Q*_*d*_ flow rates, the minimum value of the MFA appears at P3 in the middle of the impeller, and the MFA is 0.031 and 0.0704, respectively. Compared with the DC, at the optimum point of the RPC, the MFA of monitoring points P2 and P3 decreased by 76.58% and 68.33%, respectively, and the MFA of monitoring point P4 increased by 43.84%.

Figure 15c shows that when the pump system is under the RPGC, except for monitoring point P2, the MFA of the monitoring point increases with increasing flow rate. However, the location of the maximum value of the MFA at each monitoring point has a certain randomness. Under the flow condition of 1.30*Q*_*d*_, the maximum value of the MFA appears at the impeller outlet monitoring point P2 under reverse operation, and the MFA is 0.092. Under the flow conditions of 1.62*Q*_*d*_ and 1.94*Q*_*d*_, the maximum MFA appears at monitoring point P3 in the middle of the impeller, and the MFA is 0.176 and 0.38, respectively. The minimum value of the MFA always appears at impeller inlet monitoring point P4 in reverse operation, and the minimum MFA values are 0.252, 0.118 and 0.019 at flow rates of 1.30*Q*_*d*_, 1.62*Q*_*d*_ and 1.94*Q*_*d*_, respectively. Compared with the DC, the MFA of monitoring points P2, P3 and P4 increased by 24.16%, 77.71% and 139.92%, respectively, at the optimum point of the RPGC.

### Frequency division components of pressure pulsation

**Figure 16:**
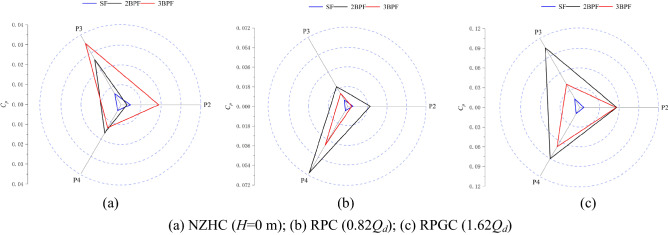
Pressure fluctuation of frequency components at impeller monitoring points under special utilization conditions.

Figure 16 shows the amplitude of the SF and the higher harmonic components of the BPF at each monitoring point under special utilization conditions. Figure 16 shows that the pressure pulsation of each monitoring point in the impeller area is mainly composed of 2BPF and 3BPF frequency components except the main frequency, and there is a certain pulsation at the SF frequency component. The occurrence of the SF frequency component is mainly related to mechanical defects, such as axial imbalance of the pump system.

Figure 16a shows that when *H* = 0 m, the *C*_*p*_ amplitudes of the SF frequency, 2BPF frequency and 3BPF frequency at impeller inlet monitoring point P2 are 0.0038, 0.0028 and 0.018, respectively, which are 7.60%, 5.60% and 36.00% of the MFA, respectively. The *C*_*p*_ amplitudes of the SF frequency, 2BPF frequency and 3BPF frequency at impeller outlet monitoring point P4 are 0.004, 0.016 and 0.012, respectively, which are 25.00%, 100.00% and 75.00% of the MFA, respectively.

Figure 16b shows that at the optimum point of the RPC, the *C*_*p*_ amplitudes of the SF frequency, 2BPF frequency and 3BPF frequency at impeller inlet monitoring point P4 are 0.005, 0.070 and 0.043, respectively, which are 7.14%, 100% and 61.43% of the MFA, respectively. The *C*_*p*_ amplitudes of the SF frequency, 2BPF frequency and 3BPF frequency at impeller outlet monitoring point P2 are 0.006, 0.021 and 0.005, respectively, which are 18.84%, 65.93% and 15.70% of the MFA, respectively.

Figure 16c shows that at the optimum point of the RPGC, the *C*_*p*_ amplitudes of the SF frequency, 2BPF frequency and 3BPF frequency at impeller inlet monitoring point P4 are 0.012, 0.090 and 0.071, respectively, which are 10.21%, 76.56% and 60.39% of the MFA, respectively. The *C*_*p*_ amplitudes of the SF frequency, 2BPF frequency and 3BPF frequency at impeller outlet monitoring point P2 are 0.009, 0.056 and 0.056, respectively, which are 5.33%, 33.16% and 33.16% of the MFA, respectively.

### Peak-to-peak value of the pressure pulsation signal

To monitor the operational stability of the pump system more intuitively, the concept of the peak value of pressure fluctuation is introduced. The peak-to-peak value of pressure pulsation represents the variation range of pulsation signal in a period, namely, the difference between the maximum value and the minimum value of the signal in a period. Based on the interval estimation of the pressure pulsation signal with a 97% confidence interval, the peak-to-peak value of pressure pulsation at each monitoring point under special working conditions is obtained in this paper. Figure 14 shows the peak value of pressure fluctuation at each monitoring point under special utilization conditions. To simplify the expression, the peak-to-peak value of pressure pulsation is referred to as PPV.

**Figure 17:**
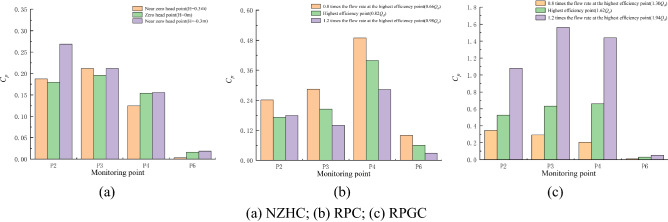
Peak-to-peak value of pressure pulsation under special utilization conditions.

Figure 17a shows that under the NZHC, with the change in head, the variation trend of the PPV at each monitoring point has no obvious regularity, and the PPV at each monitoring point in the impeller area is relatively close. Compared with the *H* = 0 m condition, the PPV at the impeller inlet increased by 5.93%, and the PPV at the impeller outlet decreased by 16.66% at the *H* = 0.30 m condition. The PPV at the impeller inlet decreased by 18.12% and at the impeller outlet increased by 1.34% at the *H* = − 0.30 m condition.

Figure 17b shows that when the pump system is under the RPC, the change trend of the PPV with flow rate at each monitoring point is basically the same. Compared with the optimum point 0.82*Q*_d_ flow condition, the PPV at the impeller inlet increased by 19.48% and the PPV at the impeller outlet increased by 42.86% at 0.66*Q*_d_ flow condition. The PPV at the impeller inlet decreased by 32.68% and at the impeller outlet increased by 7.14% at a 0.98*Q*_d_ flow rate.

Figure 17c shows that when the pump system is under the RPGC, the PPV of each monitoring point increases significantly with increasing flow rate. Compared with the optimum point of the 1.62*Q*_*d*_ flow condition, the PPV at the impeller inlet decreased by 70.97% and the PPV at the impeller outlet decreased by 34.34% under the flow condition of 1.30*Q*_*d*_. At 1.94*Q*_*d*_ flow rate, the PPV at the impeller inlet increased by 125.81%, and the PPV at the impeller outlet increased by 105.87%.

**Figure 18:**
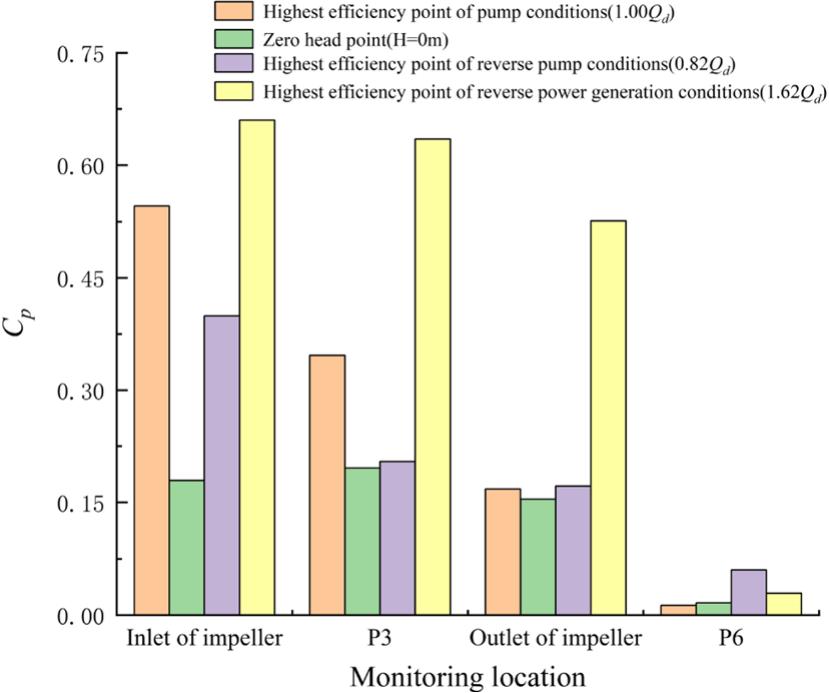
Comparison of the peak-to-peak value of pressure pulsation between the DC and special utilization conditions.

Figure 18 shows the comparison of the peak-to-peak value of pressure pulsation between the DC and special utilization conditions. Figure 18 shows that compared with the DC, the PPV under the NZHC and RPC is relatively small, and the PPV under the RPGC is relatively large. Compared with the DC, the PPV of the impeller inlet decreased by 67.16% and the PPV of the impeller outlet decreased by 8.14% under the *H* = 0 m condition. At the optimum RPC point, the PPV at the impeller inlet increased by 122.61%, and the PPV at the impeller outlet increased by 11.37%. At the optimum point of the RPGC, the PPV at the impeller inlet increased by 65.34%, and the PPV at the impeller outlet increased by 206.40%.

## Numerical simulation

### Grid generation

**Figure 19:**
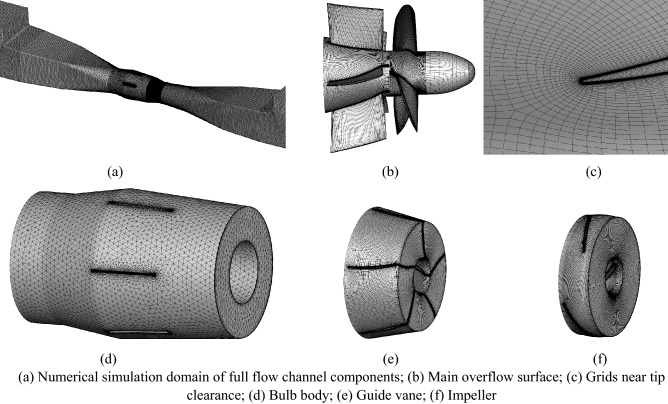
Grid diagram of the final grid scheme.

In the numerical simulation, the quality and quantity of the mesh have a great influence on the accuracy of the numerical simulation results^8,21^. Considering the good adaptability and high mesh quality of the three-dimensional hexahedral structure mesh, the computational domain except the bulb body uses ANSYS-ICEM meshing software for the dissection of the structured mesh. An O-grid is used to divide the inlet and outlet channels to increase the mesh density of the boundary layer. In addition, the grid near the wall is encrypted to accurately capture the data near the wall. Since the SST *k–ω* turbulence model equation used in this paper contains analytical expressions, the *ω* equation itself can be directly used to integrate over the viscous bottom layer by mixing the viscous bottom layer equation with the logarithmic layer equation to generate a y + -insensitive treatment model^22^. This processing mode can realize automatic switching from the wall function to the low Reynolds number model; that is, when the near-wall area mesh is relatively fine, it automatically switches to the low Reynolds number model, and when the near-wall area mesh is relatively coarse, it automatically calls the wall function. In CFX, this is called the automatic wall treatment model. Compared with the wall function method, the automatic wall treatment model of the SST *k–ω* turbulence model in CFX greatly reduces the sensitivity to the mesh at the sidewalls, and the impeller wall boundary layer thickness y + is controlled within 10 to ensure good computational accuracy. The vast majority of the grids in the computational domain of this paper have y + values within 10, which can meet the computational requirements of the turbulence model. Before the numerical calculation, five grid numbers were chosen to evaluate the grid independence. The results of the grid independence test are shown in Table 4. When the grid number is greater than 5.09 × 10^6^, the fluctuations in the flow, head and torque of the pump system under DC tend to be stable. After integrating the computational resources and grid accuracy, the final grid number of 6.26 × 10^6^ million was selected for this numerical calculation. Table 5 shows the detailed grid parameters of the final grid scheme. The grid diagram of the final grid scheme is shown in Fig. 19.

**Table 4:** Grid independence test.

Scheme	Number of grids	*H* (*m*)	*η* (%)	*M* (N m)
S1	2.43 × 10^6^	2.13	67.56	59.96
S2	3.89 × 10^6^	2.06	67.87	57.38
S3	5.09 × 10^6^	1.97	68.03	54.75
S4	6.26 × 10^6^	1.95	68.12	54.12
S5	7.24 × 10^6^	1.96	68.15	54.37

**Table 5:** Detailed grid parameters of the final grid scheme.

Domain	Mesh characteristics	Unit	Grid scheme S5
Inlet channel	Grid number	/10^6^	0.92
	Min. angle	/°	35
	Grid quality		0.55
Impeller	Grid number	/10^6^	2.21
	Min. angle	/°	28
	Grid quality		0.50
Guide vanes	Grid number	/10^6^	1.26
	Min. angle	/°	29
	Grid quality		0.50
Bulb body	Grid number	/10^6^	1.15
	Min. angle	/°	39
	Grid quality		0.45
Outlet channel	Grid number	/10^6^	0.73
	Min. angle	/°	37
	Grid quality		0.65

### Numerical methods and boundary conditions

The numerical simulations were performed based on the commercial fluid calculation software ANSYS CFX 17.0. The numerical simulation uses the continuity equation and Navier–Stokes equation as the governing equations of the flow. The governing equation of flow is as follows^23^:

6 $$\frac{\partial \rho }{{\partial t}} + \frac{{\partial (\rho u_{i} )}}{{\partial x_{i} }} = 0$$

where $i$ = 1, 2, 3 is the dumb index, namely, the repeated index. $u_{i}$ is the fluid velocity component in the coordinate direction. $\rho$ is the fluid density. $t$ is time.

7 $$\frac{{\partial (\rho u_{i} )}}{\partial t} + \frac{{\partial (\rho u_{i} u_{j} )}}{{\partial x_{i} }} = - \frac{\partial p}{{\partial x_{i} }} + \frac{\partial }{{\partial x_{j} }}\left[ {\mu (\frac{{\partial u_{i} }}{{\partial x_{j} }} + \frac{{\partial u_{j} }}{{\partial x_{i} }})} \right] + S_{mi}$$

where $p$ is the static pressure strength. $S_{mi}$ is the generalized source term of the momentum equation, including gravity and multiphase flow interphase force.

8 $$\frac{{\partial (\rho {\varvec{u}})}}{\partial t} + \nabla \cdot \, (\rho {\varvec{uu}}) = - \nabla p + \nabla \cdot \, \left[ {\nabla {\varvec{u}} + (\nabla {\varvec{u}})^{ \, T} } \right] + S_{m}$$

where ${\varvec{u}}$ is the fluid velocity vector. $\nabla {\varvec{u}}$ is the gradient of velocity vector ${\varvec{u}}$. ${\varvec{uu}}$ is the second-order tensor formed by the integration of two vectors, representing the momentum flux. $\nabla \cdot \, (\rho {\varvec{uu}})$ is the first-order tensor, indicating the divergence of momentum flux. $S_{m}$ is the generalized source term of the body force vector.

To close the governing equations, the turbulence model is needed. This numerical simulation selects the shear-stress transport (SST) *k*–*ω* turbulence model, and the SST *k*–*ω* turbulence model equations are as follows^23^:

9 $$\frac{{\partial (\rho_{{\text{m}}} k)}}{\partial t} + \frac{{\partial \left( {\rho_{{\text{m}}} u_{j} k} \right)}}{{\partial x_{j} }} = \frac{\partial }{{\partial x_{j} }}\left[ {\left( {\mu_{{\text{m}}} + \frac{{\mu_{t} }}{{\sigma_{k} }}} \right)\frac{\partial k}{{\partial x_{j} }}} \right] + P_{k} - \beta^{*} \rho_{{\text{m}}} \omega k$$

10 $$\frac{{\partial (\rho_{{\text{m}}} \omega )}}{\partial t} + \frac{{\partial \left( {\rho_{{\text{m}}} u_{j} \omega } \right)}}{{\partial x_{j} }} = \frac{\partial }{{\partial x_{j} }}\left[ {\left( {\mu_{{\text{m}}} + \frac{{\mu_{t} }}{{\sigma_{\omega } }}} \right)\frac{\partial \omega }{{\partial x_{j} }}} \right] + \frac{\alpha \omega }{k}P_{k} - D_{\omega } + Cd_{\omega }$$

11 $$\left. {\begin{array}{*{20}l} {\mu_{t} = \frac{{\rho_{{\text{m}}} \alpha_{1} k}}{{\max \left( {\alpha_{1} \omega ,SF_{2} } \right)}}} \hfill \\ {D_{\omega } = \beta \rho_{{\text{m}}} \omega^{2} ,C = 2\rho_{{\text{m}}} \left( {1 - F_{1} } \right)} \hfill \\ {d_{\omega } = \frac{1}{{\omega \sigma_{\omega 2} }}\frac{\partial k}{{\partial x_{j} }}\frac{\partial \omega }{{\partial x_{j} }}} \hfill \\ \end{array} } \right\}$$

where $k$ is the turbulent kinetic energy. ${\upomega }$ is the turbulence frequency. $P_{k}$ is the production rate of turbulence. $\rho_{m}$ is the mixture density, kg/m^3^. $u_{j}$ is the velocity component in the j direction. $\mu_{t}$ is the turbulence viscosity, and $\mu$ is the dynamic viscosity, Pa s. $F_{1}$ and $F_{2}$ are mixed functions. $\beta^{*}$, ${\upbeta }$, ${\upalpha }$, $\alpha_{1}$, $\alpha_{k}$, $\sigma_{\omega }$, $\sigma_{\omega 2}$ are all empirical coefficients. $S$ is the invariant of the strain rate. $D_{\omega }$ is the dissipation term in the ${\upomega }$-equation. $Cd_{\omega }$ is the cross-diffusion term in the SST *k–ω* model.

Extensions are added at the inlet and outlet of the axial flow pump system. The no slip assumption is adopted for each flow-through component. The boundary condition of the inlet selects mass flow, the boundary condition of the outlet selects opening, and the relative pressure is set to 0 Pa. The nonconstant numerical simulation uses the results of the constant numerical simulation as the initial condition. The freezing rotor method is used to address the rotating impeller and stationary parts in unsteady numerical simulation. The convection term is solved by a high-resolution scheme, and the transient term is solved by second-order backwards Euler. The time step is set to 0.0005 s, and the total calculation time is 0.48 s (8 impeller rotation cycles).

## Numerical simulation results and analysis

### Comparison of external characteristics

**Figure 20:**
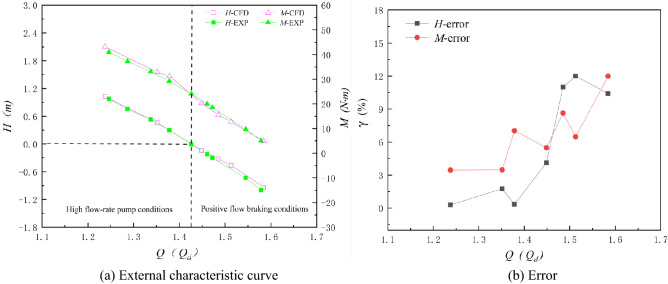
Comparison of numerical simulation results and experimental results of the NZHC.

**Figure 21:**
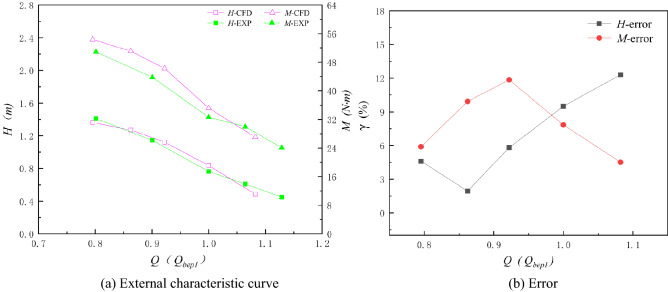
Comparison of numerical simulation results and experimental results of the RPC.

**Figure 22:**
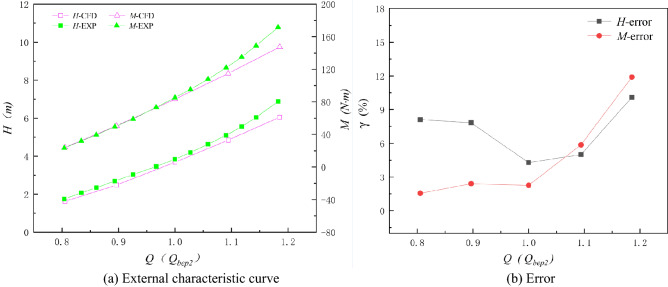
Comparison of the numerical simulation results and experimental results of the RPGC.

In this paper, the axial flow pump systems under three special utilization conditions are numerically calculated. To compare the numerical results for different special operating conditions more independently with the experimental results, the flow rates of the axial flow pump system under three special operating conditions are treated independently and are dimensionless. Figures 20, 21 and 22 show the comparison between the numerical simulation results and experimental results under three special utilization conditions. *Q*_bep1_ in Fig. 21 is the flow rate corresponding to the optimum point of the RPC, and *Q*_bep2_ in Fig. 22 is the flow rate corresponding to the optimum point of the RPGC. The external characteristic curves of the numerical simulation and the experiment are highly consistent in trend under the NZHC and RPGC. The trends of the external characteristic curves of the numerical simulation and experiments under the RPC are roughly consistent, but the agreement in details is poor. From the error analysis of the numerical simulation results and the experimental results, it can be found that the error of the numerical simulation fluctuates around approximately 5% in most cases, and the maximum error is within 13%.

### Flow field analysis in the pump

**Figure 23:**
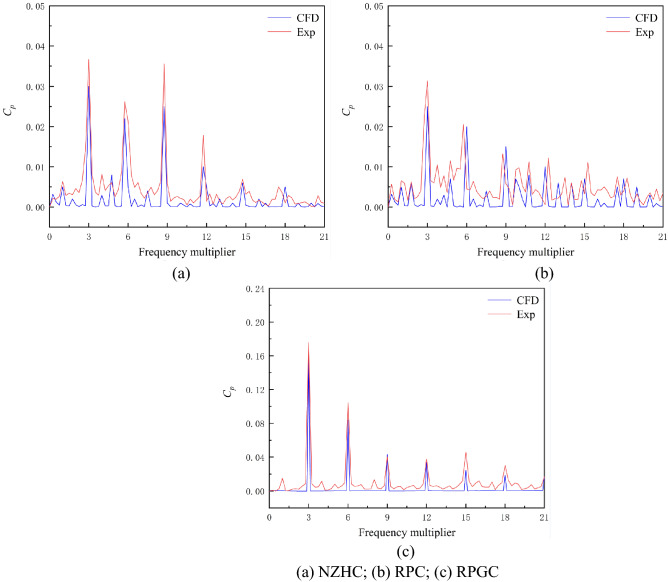
Comparison of the numerical simulation value and experimental value of the pressure pulsation.

To further explain and analyse the pressure pulsation in the pump under special utilization conditions combined with the flow field in the pump, the unsteady numerical simulation of the pump system under three special utilization conditions is carried out in this paper. Figure 23 shows the comparison between the pressure fluctuation in the pump obtained by unsteady numerical simulation and the pressure fluctuation in the pump measured by experiment. Figure 23 shows that under the NZHC and RPGC, the frequency components of the pressure pulsation signal obtained by the numerical simulation and experiment are basically the same, and the amplitude difference is small. Under the RPC, the frequency component of the pressure fluctuation signal obtained by numerical simulation and experiment has a certain error, and the amplitude also has a certain difference. The reason is that there is a serious unstable flow in the pump under the reverse pump condition, which leads to a decrease in the reliability of the numerical simulation. In general, the pressure pulsation obtained by the unsteady numerical calculation can reflect the law of the pressure pulsation measured by the experiment, and the internal flow field obtained by the unsteady numerical calculation can be used to further explain and analyse the pressure pulsation in the pump.

**Figure 24:**
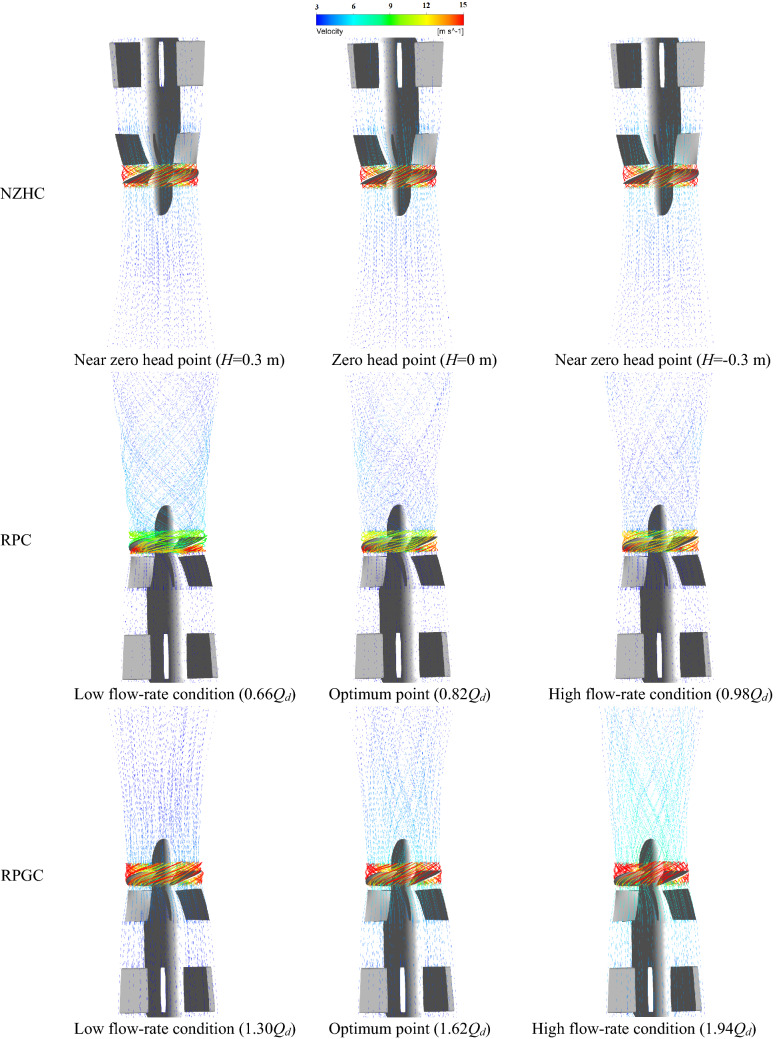
Internal flow line of the pump system under special utilization conditions.

Figure 24 is the internal streamline of the pump system under special utilization conditions^24–27^. Figure 24 shows that under the RPC, the flow pattern in the pump system is disordered, and a serious flow separation phenomenon occurs after the flow passes through the impeller. Under the 0.66*Q*_*d*_ flow condition and 0.82*Q*_*d*_ flow condition, the spiral flow is full of channels. This is also consistent with the fact that there is a large amount of pulsation in the low-frequency and high-frequency areas of the pressure pulsation spectrum of the RPC in the pressure pulsation test. Under the NZHC and RPGC, the flow pattern inside the pump system is relatively good, the flow line distribution behind the impeller is relatively smooth and regular, and the flow in the flow channel shows a certain symmetry. This is also consistent with the regular pressure pulsation waveform and relatively simple pressure pulsation signal components of each monitoring point under the NZHC and RPGC in the pressure pulsation test.

**Figure 25:**
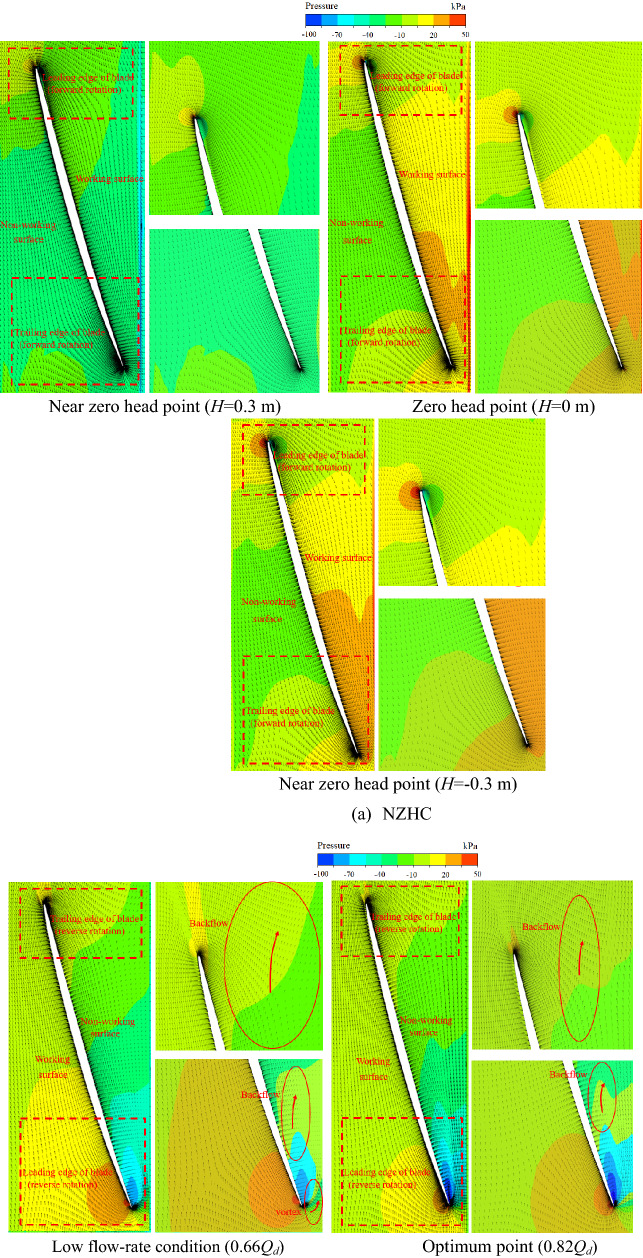

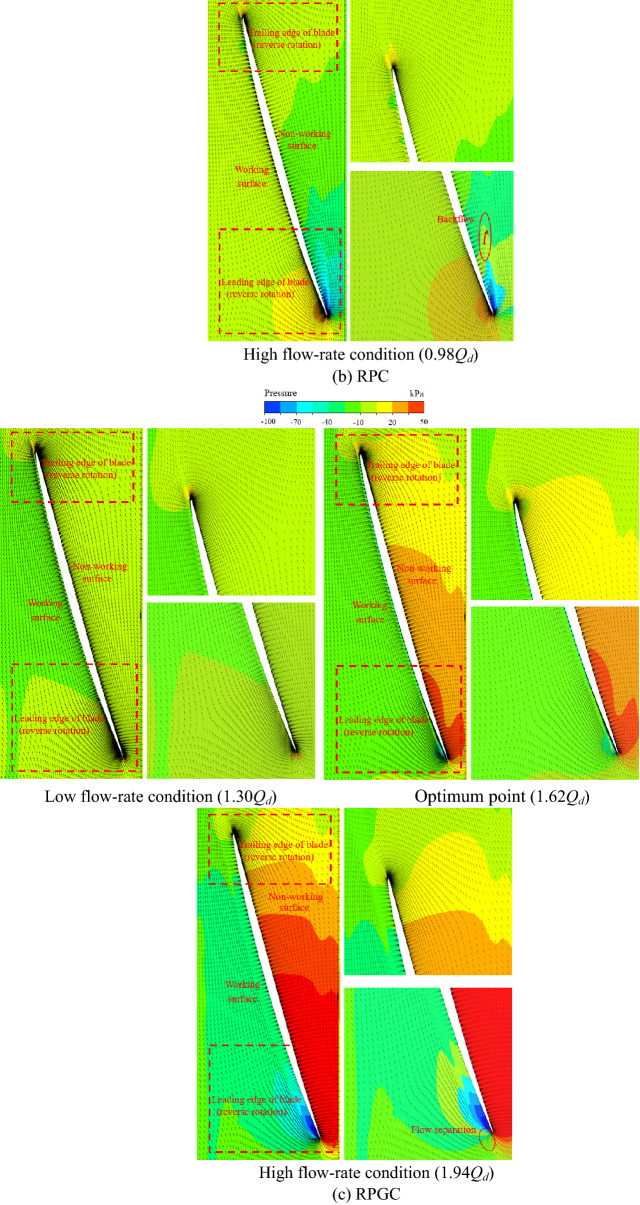
Pressure distribution and velocity vector diagram of 0.99 times the blade height in the pump section (span = 0.99).

Figure 25 is 0.99 times the blade height (close to the wall of the pump system) of the pump pressure distribution and velocity vector. Figure 25 shows that under the NZHC, the flow rate of the pump system is large, the angle between the relative velocity of the liquid flow and the circumferential direction is increased, and the blade angle is not changed, resulting in a decrease in the blade airfoil angle. There is no obvious vortex or backflow zone in the blade nonworking face. The velocity and pressure distribution are uniform, and the collision and diffusion of the flow in the pump are not too serious. This result is close to the internal flow field obtained by Wang’s numerical simulation of an axial flow pump system under an NZHC^1^. This is also consistent with the smaller PPV of each monitoring point in the impeller area under the NZHC in the pressure pulsation test. Under the RPC, there is a large pressure gradient in the leading edge of the blade, and a large range of reflux areas appears in the nonworking face of the blade. The fluid in the pump shows the opposite movement trend with the inlet inflow. The adverse flow, such as reflux and secondary flow in the pump, leads to the complex pressure pulsation signal in the pump. In the pressure pulsation test, the complex pressure pulsation signal components of each monitoring point under the RPC also verify this. With the increase in the flow rate under RPC, the range of the recirculation zone gradually decreases, and the flow pattern at the impeller inlet gradually improves. This is also consistent with the situation that the component of the pressure pulsation signal tends to be simple, and the pulsations in the low-frequency region and high-frequency region disappear at monitoring point P6 in the pressure pulsation test under a large flow rate RPC. Under the RPGC, when the pump system runs at 1.30*Q*_*d*_ and 1.62*Q*_*d*_ flow conditions, the flow state in the pump is good, and no flow separation is found in the nonworking face of the impeller. When the flow rate increases to 1.94*Q*_*d*_, a large pressure gradient appears at the leading edge of the impeller, and local flow separation occurs at the nonworking face of the impeller. In general, the internal flow pattern of the axial flow pump system is better when reversing power generation, which is consistent with the conclusion of Qian's research on reversing power generation of a small axial flow pump^11^.

## Conclusion

To explore the hydrodynamic characteristics of an axial flow pump system under special utilization conditions, a high-precision full-feature test bench for the axial flow pump system is established in this paper. For the first time, an energy characteristics experiment and a pressure fluctuation measurement in the pump are carried out for a large axial flow pump system model under the NZHC, RPC and RPGC. Then, ANSYS CFX software is used to solve the continuous equation and Reynolds average Navier–Stokes equation, combined with the SST *k–ω* turbulence model, and the characteristic curve and internal flow field of the pump system under special conditions are obtained. Finally, the numerical simulation results are compared with the experimental results. The main conclusions are as follows:

1. The pressure pulsation amplitude of the NZHC is small, and the maximum value of the MFA in the impeller area always appears at the impeller inlet. Compared with the DC, the MFA of monitoring points P2, P3 and P4 at H = 0 m decreased by 62.99%, 63.00% and 66.86%, respectively. The PPV at the impeller inlet decreased by 67.16%, and the PPV at the impeller outlet decreased by 8.14%. The velocity and pressure distribution in the pump are uniform, and the flow collision, reflux and diffusion in the pump are not too serious.
2. The composition of the pressure fluctuation signal of the RP is complex, and the high-order harmonic components of the blade frequency are more obvious. The maximum MFA in the impeller area always appears at the inlet of the impeller. Compared with the DC, under the optimum point of the RPC, the MFA of monitoring points P2 and P3 decreased by 76.58% and 68.33%, the MFA of monitoring point P4 increased by 43.84%, the PPV of the impeller inlet increased by 122.61%, and the PPV of the impeller outlet increased by 11.37%. The unstable flow phenomenon in the pump is obvious. There is a large pressure gradient in the leading edge of the blade, and a large range of backflow zones appear in the nonworking face of the blade.
3. The waveform of pressure pulsation of the RPGC has good regularity and periodicity, and the minimum value of the MFA always appears at the inlet of the impeller. Compared with the DC, the MFA of monitoring points P2, P3 and P4 increased by 24.16%, 77.71% and 139.92% under the optimum point of the RPGC, the PPV at the impeller inlet increased by 65.34%, and the PPV at the impeller outlet increased by 206.40%. The flow state in the pump is good, and no obvious flow separation phenomenon is found in the nonworking face of the impeller.

The pressure pulsation of the pump system running near the zero head will not affect the safe and stable operation of the pump system. This result is also consistent with the findings obtained from field tests conducted by Wang et al.^1^ on an inclined axial flow pump system under an NZHC. When the pump system reverses pumping, there is a large amount of pulsation in the low- and high-frequency regions of the pressure pulsation signal, which is very likely to affect the safe and stable operation of the unit. This is different from the conclusion obtained by Ma et al.^3^ after studying the hydrodynamic characteristics of a bidirectional pump under the RPC. This is because the object of Ma is an axial flow pump designed for bidirectional operation, and the object of this paper is an axial flow pump designed for unidirectional operation. The composition of the pressure fluctuation signal of the axial flow pump system in reverse power generation is simple and has little influence on the safe operation of the pump system. This is also consistent with the conclusion of excellent hydraulic efficiency and better internal flow regime for axial flow pumps operating for reverse power generation, as indicated by Qian et al.^11^.

The current work mainly uses experimental methods to reveal the hydrodynamic characteristics of the pump system, especially the pressure pulsation characteristics under special utilization conditions. At the same time, the possibility of multifunctional utilization and safety stability of the axial flow pump system are evaluated by comparing the special working condition with the design condition. The research results can provide an important reference for the safe and stable operation of a low-lift axial flow pump station system under special utilization conditions. However, how to eliminate or improve the pressure pulsation in the pump under special conditions has not been well solved. In further research, more physical analysis of the pump system under special conditions should be considered based on the CFD method to reveal the damage mechanism of pressure pulsation on the pump system under special conditions.

## Nomenclature

*C*_p_: Pressure pulsation coefficient
*f*: Frequency (s^−^^1^)
F: Frequency after Fourier transform (s^−^^1^)
*g*: Local acceleration of gravity (m/s^2^)
*H*_exp_: Experimental head (m)
*M*: The impeller torque (N m)
*n*: Rated speed (r/min)
*N*_*F*_: Multiple of rotational frequency
*P*: Transient pressure (Pa)
$\overline{p}$: Average pressure (Pa)
*Q*: Flow rate of the model pump system (m^3^/s)
*Q*_*d*_: Designed flow
*t*: Time (s)
*u*_2_: Circumferential velocity of impeller outlet (m/s)
*ρ*: The density of flow (kg/m^3^)
*ω*: Angular velocity of the impeller (rad/s)
*η*: Efficiency (%)
*η*_exp_: Experimental efficiency (%)
γ: Error

## Abbreviations

CFD: Computational fluid dynamics
SF: Shaft frequency
SST: Shear-stress transport
BPF: Impeller rotation frequency
DC: Design condition of pump system
NZHC: Near zero head working condition
RPC: Reverse pump condition
RPGC: Reverse power generation condition
MFA: Main frequency amplitude
PPV: Peak-to-peak value of pressure pulsation
